# Heart rate variability and cardiac repolarization after exposure to zinc oxide nanoparticles in healthy adults

**DOI:** 10.1186/s12995-020-00255-2

**Published:** 2020-02-28

**Authors:** Assem Aweimer, Birger Jettkant, Christian Monsé, Olaf Hagemeyer, Vera van Kampen, Benjamin Kendzia, Vitali Gering, Eike-Maximilian Marek, Jürgen Bünger, Andreas Mügge, Thomas Brüning, Rolf Merget

**Affiliations:** 1 Department of Cardiology and Angiology Bergmannsheil University Hospital, Ruhr-Universität Bochum, Bürkle-de-la-Camp-Platz 1, 44789 Bochum, Germany; 2 Institute for Prevention and Occupational Medicine of the German Social Accident Insurance, Institute of the Ruhr-Universität Bochum (IPA), Bürkle-de-la-Camp-Platz 1, 44789 Bochum, Germany

**Keywords:** Zinc oxide, Nanoparticles, Heart rate variability, Cardiac repolarization

## Abstract

**Background:**

Exposure to airborne zinc oxide (ZnO) particles occurs in many industrial processes, especially in galvanizing and welding. Systemic inflammation after experimental inhalation of ZnO particles has been demonstrated previously, but little is known about the impact on the cardiovascular system, particularly on the autonomic cardiac system and the risk of arrhythmias. In this study we investigated the short-term effects of ZnO nanoparticles on heart rate variability (HRV) and repolarization in healthy adults in a concentration-dependent manner at rest and during exercise in a controlled experimental set-up.

**Methods:**

Sixteen healthy subjects were exposed to filtered air and ZnO particles (0.5, 1.0 and 2.0 mg/m^3^) for 4 h, including 2 h of cycling at low workloads. Parameters were assessed before, during, immediately after, and about 24 h after each exposure. For each subject, a total number of 46 10-min-sections from electrocardiographic records were analyzed. Various parameters of HRV and QT interval were measured.

**Results:**

Overall, no statistically significant effects of controlled ZnO inhalation on HRV parameters and QT interval were observed. Additionally, a concentration-response was absent.

**Conclusion:**

Inhalation of ZnO nanoparticles up to 2.0 mg/m^3^ for 4 h does not affect HRV and cardiac repolarization in healthy adults at the chosen time points. This study supports the view that cardiac endpoints are insensitive for the assessment of adverse effects after short-term inhalation of ZnO nanoparticles.

## Background

Zinc and zinc compounds like zink oxide (ZnO) occur in many industrial processes and especially galvanizing and welding workers are exposed to nano-sized ZnO particles. Inhalation of ZnO particles have previously been demonstrated to cause systemic inflammatory responses named “metal fume fever” or “zinc fever” [1]. In general, there is a latency of few hours until the onset of symptoms. Experimental inhalation studies investigating zinc containing welding fumes showed that inflammatory effects may occur with ZnO concentrations below 2.0 mg/m^3^ ZnO [2, 3]. However, one study with low concentrations of pure ZnO (0.5 mg/m^3^) reported no effects in 12 subjects after inhalation for 2 h at rest [4], including no effects on HRV. In a rat experiment, cardiac inflammation and the development of fibrosis 7 days after exposure to ZnO nanoparticles was observed [5]. Recently, we reported flu-like symptoms, fever and an increase of inflammatory markers in blood after exposure to inhaled ZnO nanoparticles at or above 1 mg/m^3^ [6].

Derived from our recent publication [6] it has been pointed out that systemic inflammation after ZnO inhalation could lead to long-term cardiac effects [7], whereby the role of autonomic imbalance and its relationship to systemic inflammation remains unclear. Furthermore, respiratory reflexes which affect the autonomic nervous system may lead to alterations in heart rate, HRV and arrhythmia [8].

In many panel studies HRV was taken as a parameter to measure the effects on the autonomic cardiac system, however due to methodological issues like small numbers of subjects and multiple testing the conclusions of these studies are considered limited [9]. Inhalation studies that use more than one concentration step are extremely rare and there is no study which has shown concentration-dependent effects on HRV or repolarization after inhalation of hazardous substances.

In this study we tested the hypothesis that acute inhalation of ZnO nanoparticles at different concentrations causes concentration-dependent changes of HRV and repolarization in healthy adults in a controlled experimental set-up.

## Methods

### Study design and experimental set-up

The detailed methodology and experimental set-up including a graphical timeline was described recently [6]. Briefly, subjects were exposed four times for 4 h with 2 weeks intervals in an exposure unit at our institute [10] to each exposure scenario: filtered air (sham) and 3 different ZnO particle concentrations (0.5, 1.0 and 2.0 mg/m^3^). ZnO particle synthesis was based on pyrolysis of atomized aqueous zinc formate solutions with a hydrogen-oxygen flame. The particle size of the generated primary particles was determined with scanning electron microscopy (SEM, model JSM-7500F, JEOL Ltd., Tokyo, Japan) and was about 10 nm [11]. Depending on the ZnO concentration the primary particles formed aggregates and agglomerates in a range from 48 nm (0.5 mg/m^3^ ZnO) to 86 nm (2.0 mg/m^3^ ZnO), determined with a scanning mobility particle sizer (SMPS, model 3080, TSI Inc., Shoreview MN, USA, equipped with a long differential mobility analyzer and a butanol condensation particle counter, model 3776, TSI Inc.) [10]. Measurements on airborne ZnO particles with an electrometer (Modell 3068B, TSI Inc., Shoreview MN USA) could not detect any electric charge.

X-ray powder diffraction of ZnO particles, that were sampled via thermophoresis, was determined using a diffractometer from Stoe with a Bragg-Brentano geometry (XRD, model Stadi P with Co Anode and scintillation counter, Stoe & Cie GmbH, Darmstadt, Germany). Comparing this with standard data, it was observed that all the peaks were matched with the standard data of hexagonal phase of zinc oxide (JCPDS card no. 36–1451).

An elementary analysis of the ZnO particles (Mikroanalytisches Labor Pascher, Remagen, Germany) yielded a purity of 99.7%. The specific surface area as determined by a BET device (BET, model Gemini VII 2390a, Micromeritics GmbH, Aachen, Germany) was 20.2 g/m^3^.

A ceiling fan was used to homogenize the freshly generated ZnO nanoparticle atmospheres in the exposure unit [12]. Briefly, constant target concentrations with 0.5, 1.0 and 2.0 mg/m^3^ ZnO were planned. Sham exposures (0 mg/m^3^ ZnO) were also performed with the flame generator operated with purified water without zinc salt. The purity of the airborne ZnO was 99.71%. The air exchange rate was set at 12 per hour (360 m^3^/h) with a room temperature of 23.5 °C (+/− 0.3 °C) and a relative humidity of 47.0% (+/− 1.7%).

Potential participants were tested for their suitability to participate in the study in a baseline examination including a questionnaire, medical examination, lung function test and exercise testing. Smoker or participants with chronic diseases were excluded with the exception of sensitizations to seasonal environmental allergens. The recruitment of these volunteers was realized by advertising at universities and student residences. Sixteen healthy nonsmoking volunteers (8 women, 8 men) with a median age of 26 years (range 19–42) and a median BMI of 24 kg/m^2^ (range 19–29) participated in the study [6]. The subjects had no previous exposure to airborne zinc compounds. Standard baseline laboratory parameters were within normal ranges.

The subjects were examined during the 4 h-periods at rest and during periods of moderate physical exercise on a cycle ergometer set to 15 L/[min∙m^2^] corresponding to an individual work load of 30–96 watt. Each 30-min rest was followed by a 30-min exercise period, for four times. Exposures were randomized and double blinded, with the exception of the exposures to 2.0 mg/m^3^ ZnO, which were not blinded according to instructions by the ethics committee. Examinations were performed before, during, directly after (after about 10 min at rest), and approximately 24 h after exposure. Additionally, examinations were performed at recruitment (baseline test) and about two weeks after the last exposure (final test).

For each subject a total number of 46 10-min-sections from all electrocardiographic records were analyzed. A 10-min-section was defined as the 2nd third of every 30-min-period, thus the beginning and the end of the period were removed to ensure steady-state conditions and comparability.

### Electrocardiography (ECG)

All ECGs were recorded with a 10-lead electrode hookup and an H12+ Holter recorder (Mortara, Essen, Germany). The recording rate was updated to 1000 samples/s per channel. This device stores all leads continuously on a Compact Flash Card for a maximum of 24-h. During the hook-up an integrated LCD display and keyboard allowed quality checks e.g. of electrode impedances and system configurations. Finally the Holter ECG records were transferred and organized on a personal computer. A first processing of the prerecorded ECG data was done with the Holter analysis software H-Scribe of Mortara to identify and label arrhythmic beats or electronic artifacts. All recorded high-resolution electrocardiographic raw data files were then processed with Mortara’s SuperECG research tool [13]. As a result, a beat-to-beat analysis was obtained with enhanced accuracy. For each beat the RR interval in ms and the QT time in ms were listed for further statistical analysis. Both programs use Mortara‘s VERITAS ECG algorithms. The listed time stamps and RR interval data were then imported as a column vector in ASCII format in KubiosHRV program Ver. 2.2 [14].

HRV parameters of the RR series of each time segment were then computed e.g. linear and nonlinear time-domain, frequency-domain and power spectral density parameters. The following time domain parameters were calculated: SDNN (Standard deviation of all normal to normal beat [NN] intervals), rMSSD (square root of the mean of the sum of the squares of differences between adjacent NN intervals) and pNN50 (ratio of the number of pairs of adjacent NN intervals differing by > 50 msec to the total number of NN intervals). The spectrum powers based on fast Fourier transform (FFT) of the low frequency (LF, 0.04–0.15 Hz), and high frequency (HF, 0.15–0.4 Hz) band delivers e.g. the LF to HF ratio.

Applying Mortara’s SuperECG program the QT interval times of every beat were extracted from all ECGs. The QT interval was measured from beginning of the QRS complex up to the end of the T wave and represented the time taken for the electrical depolarization and repolarization. The mean and standard deviations QT times of all 10 min-sections were calculated. Afterwards a frequency correction of QT interval using Bazett’s formula was done. Other commonly used QT correction formulae [15] were not taken into account.

### Data analysis

Descriptive analysis was performed for each parameter stratified by exposure and time of measurements. Figures represent boxplots with medians, 25%- and 75%-quantiles as well as minimum and maximum.

Outliers were defined as values above median + 1.5 x interquartile range or values below median - 1.5 x interquartile range. In a first step, parameters assessed immediately before the exposures were compared with those after sham or ZnO exposures after the predefined time intervals. In a second step, for each of the various time points parameters were compared between exposure conditions (sham and the three ZnO concentrations). To estimate the effects of ZnO on the HRV parameters we used various generalized estimating equations (GEE) models, but the algorithm did not converge (data not shown). Therefore comparisons were performed with paired Student’s t-test for continuous variables. The problem of multiple comparisons was counteracted using the Bonferroni correction [16], by dividing the overall desired statistical significance level α by the number of hypotheses tested.

## Results

For reasons of clarity and comprehensibility all results are presented as boxplots. There were no statistical differences between males and females in any of the analyzed parameters.

### MeanRR, SDNN, rMSSD, pNN50

**Fig. 1:**
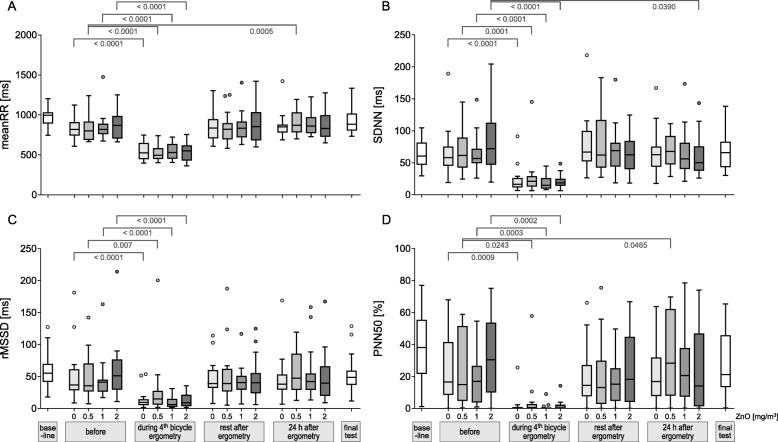
MeanRR (**a**), SDNN (**b**), rMSSD (**c**) and PNN50 (**d**) according to ZnO concentrations and time points. Differences between before exposures and the different time points with a significance level of *p* < 0.05 are indicated. A significance level of α = 0.0031 resulted after Bonferroni correction. Outliers (dots) are defined as values above median + 1.5 x interquartile range or values below median - 1.5 x interquartile range

The comparisons of the parameters meanRR, SDNN, rMSSD and pNN50 between before exposure and after sham or ZnO exposures at the predefined time points yielded significant effects only during exercise, but not after other time points (Fig. 1a–d). Few differences with a significance level of < 0.05 were considered due to multiple testing.

**Fig. 2:**
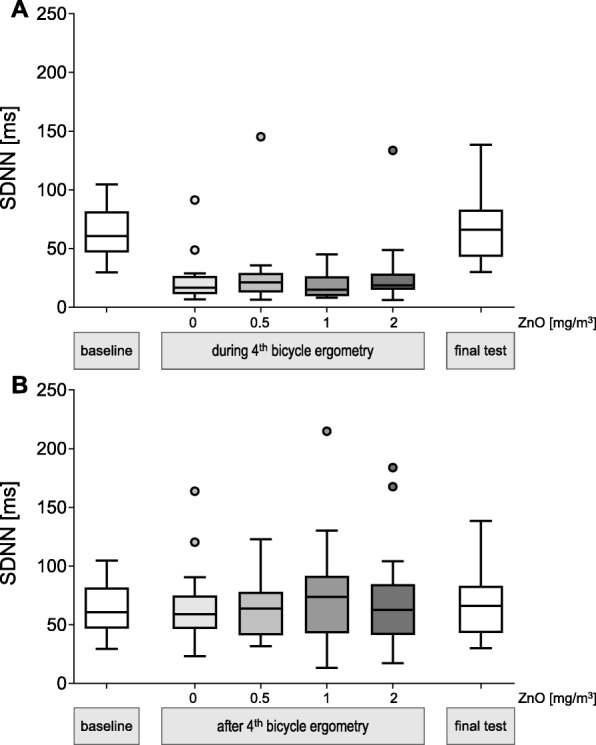
SDNN during (**a**) and at rest after 4th bicycle ergometry (**b**) after inhalation of the ZnO concentrations. No significant differences were detected between exposure conditions (all *p* values > 0.05)

When parameters were compared between exposure conditions at the various time points no significant differences were detected (this is shown representatively for SDNN for the time points ‘during exercise’ (Fig. 2a) and ‘rest after 4^th^ bicycle ergometry’ (Fig. 2b). Thus a concentration-response relationship was not observed with any parameter (other parameters and time points not shown).

### HF, LF LF/HF-ratio

**Fig. 3:**
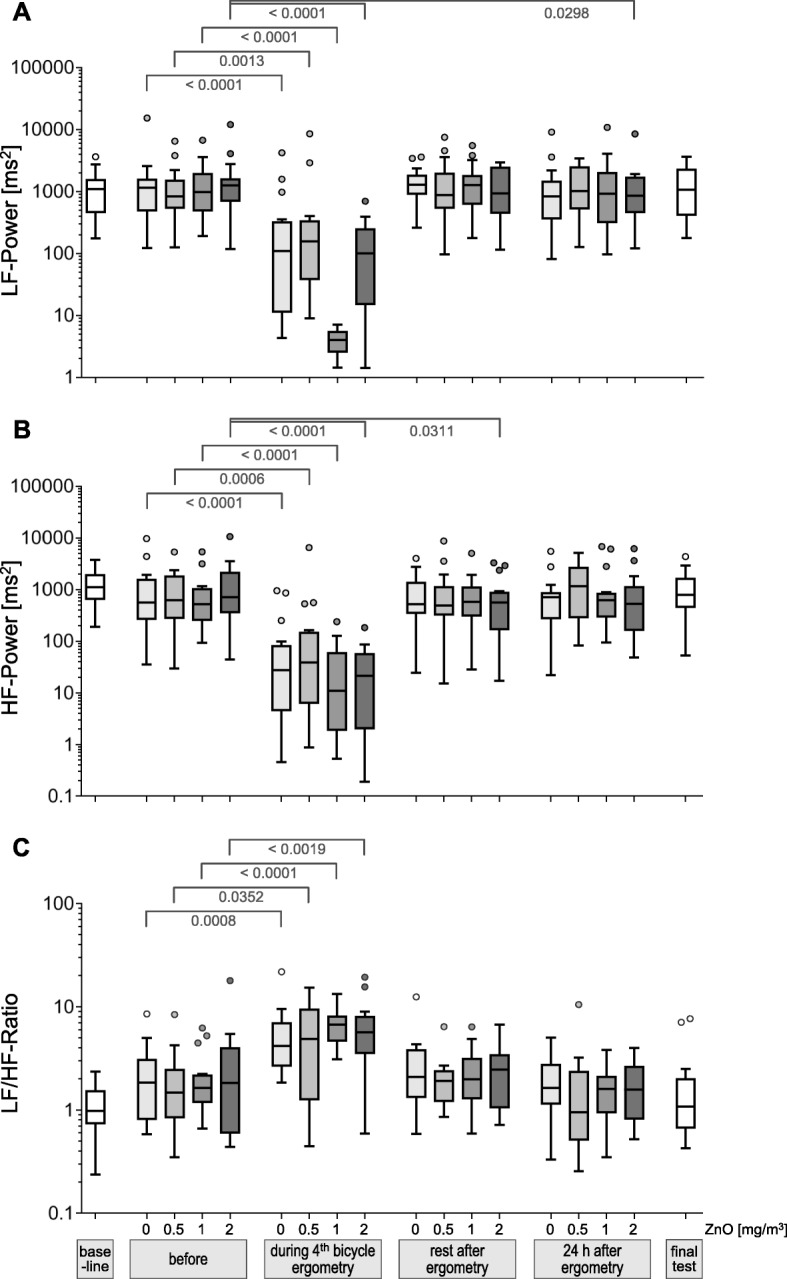
LF power (**a**), HF power (**b**) and LF/HF-ratio (**c**) according to ZnO concentrations and time points. Differences between before exposures and the different time points with a significance level of *p* < 0.05 are indicated. A significance level of α = 0.0042 resulted after Bonferroni correction. Outliers were defined as in Fig. 1

The comparisons of the parameters HF, LF and LF/HF-ratio between before exposure and after sham or ZnO exposures after the predefined time intervals yielded significant effects only during exercise, but not after other time points (Fig. 3a–c). Few differences with a significance level of < 0.05 were considered due to multiple testing.

When parameters were compared between exposure conditions at the various time points no significant differences were detected (data not shown). Thus a concentration-response relationship was not observed with any parameter.

### QTc interval

**Fig. 4:**
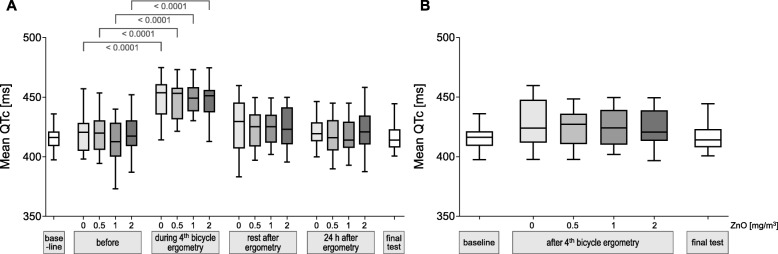
Mean QTc according to ZnO concentrations and time points. Differences between before exposures and the different time points with a significance level of *p* < 0.05 are indicated. A significance level of α = 0.0031 resulted after Bonferroni correction (Figure 4 **a**). Figure 4**b** shows mean QTc at rest after 4th bicycle ergometry after inhalation of ZnO in different concentrations. No significant differences were detected between exposure conditions (all *p* values > 0.05). Outliers were defined as in Fig. 1

The comparisons of QTc intervals between before exposure and after sham or ZnO exposures after the predefined time intervals yielded significant effects only during exercise, but not after other time points (Fig. 4a).

When QTc intervals were compared between exposure conditions at the various time points no significant differences were detected (this is shown representatively for the time point ‘rest after 4th bicycle ergometry (Fig. 4b). Thus a concentration-response relationship was not observed with this parameter.

## Discussion

To our knowledge, this is the first study investigating effects of ZnO nanoparticles on heart rate variability (HRV) and cardiac repolarization in healthy adults using a controlled inhalation set-up of three different ZnO concentrations. In all previous controlled exposure studies investigating particle inhalation effects on HRV in humans maximally two concentrations of particles like diesel exhaust [17], carbon ultrafine particles [18] or ambient particles [19] were used for defining any concentration-response relationship.

Until now, there was only one study published which investigated the effects of ZnO on HRV with a concentration of 0.5 mg/m^3^ in healthy adults. In this study no significant effects on HRV were detected [4]. A point of criticism of the study of Beckett et al. was the sole and low ZnO concentration of 0.5 mg/m^3^, which might be too low to induce significant effects on HRV.

In this study a longer duration of 4 h and higher concentrations of up to 2 mg/m^3^ were used. The maximal concentration was chosen because experimental inhalation studies with zinc containing welding fumes reported zinc-related inflammatory effects below 2 mg/m^3^. Although the concentration range of this study is lower than the exposure limit of 5 mg/m^3^ in many countries, it is closer to the proposed threshold for respirable Zn by the German MAK commission of 0.1 mg/m^3^ [20]. The 2 weeks intervals between ZnO exposures were chosen in order to minimize possible carry-over effects.

Concerning other specific effects on myocardium, cardiac fibrosis mediated by inflammation after ZnO exposure has been demonstrated in a rat model [5], but the results of this subchronic animal study are difficult to transfer to humans. As no epidemiological data are available, we cannot answer the question of cardiac sequelae after long-term exposure to ZnO.

In previous studies it has been reported that exposure to airborne particulate matter decreases HRV indices [21–25], but others observed the opposite [26–28]. However, none of these studies was designed to measure the effect on HRV by a single component of particulate matter. Although it cannot be excluded that the previously described impact of particles on HRV might result from the interplay of various particle components, we consider this hypothesis less likely. In a recently published review which focused on panel studies investigating the association between HRV and particulate matter the authors concluded that studies with apparently significant effects of particulate matter on HRV parameters were not persuading [9] due to statistical or methodological issues. Our study design ensured a high grade of methodological quality due to the experimental set-up with measurement of HRV parameters at rest and during exercise and additionally in a concentration-dependent manner. However, we were unable to find any significant concentration-dependent effects on HRV parameters after exposure to ZnO inhalation up to 2 mg/m^3^.

As an additional aim of our study we investigated the influence of ZnO particles on cardiac repolarization measuring the corrected QT interval (QTc). Our results show that there is no concentration-dependent effect on QTc at rest and during exercise. There is no comparable study, because to the best of our knowledge there has not been any study of the effects of ZnO particles on repolarization. Nonetheless, there are several studies focusing on ambient ultrafine particles and repolarization. Samet et al. reported on decreased QTc after exposure to ultrafine concentrated ambient particles [28]. In contrast, others reported about an increase of the QTc by ambient particulate matter in patients with coronary heart disease [29, 30]. Other studies did not show any significant effect on QTc, even less a concentration-dependent effect [31].

Some epidemiological human exposure studies identified susceptible populations considering HRV or QT changes [29, 30, 32]. Previously, decreasing SDDN and rMSSD in elderly subjects were reported [23, 33, 34]. Additionally, Nadziejko et al. observed an increase in the frequency of irregular and delayed beats after exposure to ambient particulate matter in older rats [35]. This age-related response to airborne particulate matter could also explain the absence of significant effects on HRV and QT interval in our young healthy study subjects.

Experimental inhalation studies in humans are complex, cumbersome and cost-intensive. Thus such studies include mostly 10–20 study subjects [36–38]. The apparently low number of subjects poses a problem if no effects are detected, as according to power analyses much higher numbers of subjects are required for parameters with high variance. This is of particular importance for cardiovascular endpoints as e.g. “normal” heart rate variability is not well known and power analyses are probably subject to substantial error. Due to the multiple time points that must be considered especially in complex parameters as e.g. heart rate variability and consecutive multiple testing, it is difficult to evaluate whether an effect after particle inhalation is “significant”. Multivariate testing is hampered by the low number of subjects, and in most studies comparisons before/after exposure as well as after fresh air/particle inhalation were performed, with the possibility that both comparisons may provide different results. We consider the number of 16 subjects in this study as a limitation, but studies with much higher numbers of subjects cannot be performed with reasonable efforts. The fact that no concentration-dependent effects were seen strengthens the interpretation that this study is indeed a negative study.

Due to rather high solubility, ZnO is not a poorly soluble particle (PSP), thus extrapolation to other particles should be made with caution. However, as we measured systemic inflammatory responses with ZnO [6], it can be concluded from this study that the cardiac parameters are less suited to assess effects after short-term ZnO inhalation. A further possible weakness of this study is the limited number of time-points after the end of exposure (directly and 24 h afterwards). However, significant systemic effects were seen after 24 h and relevant changes of HRV and repolarization should have been found at least during the last assessment 24 h after exposure.

## Conclusion

In conclusion, the results of our study suggest no significant effects of short-term ZnO inhalation on HRV and cardiac repolarization at concentrations up to 2.0 mg/m^3^ for four hours already showing systemic inflammatory effects in healthy adults, although such exposure conditions produced clear concentration-related systemic inflammatory effects.

## Data Availability

The datasets used and/or analyzed during the current study are available from the corresponding author on reasonable request.

## Abbreviations

BMI: Body Mass Index
ECG: Electrocardiography
FFT: Fast Fourier transform
HF: High frequency spectrum
HRV: Heart rate variability
LF: Low frequency spectrum
MeanRR: Mean value of all RR intervals within a segment
pNN50: Ratio of the number of pairs of adjacent NN intervals differing by > 50 msec to the total number of NN intervals
PSP: Poorly soluble particle
rMSSD: Square root of the mean of the sum of the squares of differences between adjacent NN intervals
SDNN: Standard deviation of all normal to normal beat [NN] intervals
ZnO: Zinc oxide
